# P-1062. *In vitro* Activity of Gepotidacin and Comparator Agents against a Collection of *K. pneumoniae* Urine Isolates from the United States During 2019-2022

**DOI:** 10.1093/ofid/ofae631.1251

**Published:** 2025-01-29

**Authors:** S J Ryan Arends, Renuka Kapoor, Didem Torumkuney, Rodrigo E Mendes

**Affiliations:** JMI Laboratories / Element, North Liberty, Iowa; GSK, Atlanta, Georgia; GSK, Atlanta, Georgia; JMI Laboratories, North Liberty, Iowa

## Abstract

**Background:**

Gepotidacin is a novel, bactericidal, first-in-class triazaacenaphthylene antibiotic that inhibits bacterial DNA replication by a unique mechanism of action, a distinct binding site and provides well-balanced inhibition (for most uUTI uropathogens) of two different type II topoisomerase enzymes. This study reports on the *in vitro* activity of gepotidacin and other oral antibiotics tested against contemporary *Klebsiella pneumoniae* clinical isolates collected from patients with urinary tract infections (UTI) in the United States as part of a gepotidacin global uropathogen surveillance study.

**Figure:**
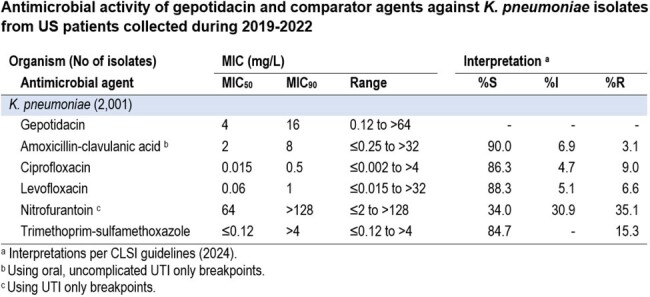

**Methods:**

A total of 2,001 *K. pneumoniae* isolates were collected during 2019-2022 from 73 medical centres located in the United States. All isolates were tested for susceptibility by CLSI methods at a central laboratory (Element Iowa City). MIC results for comparator agents were interpreted per CLSI guidelines. MIC results for oral antibiotics approved for the treatment of uncomplicated UTI. Multidrug-resistant (MDR) and extended-spectrum β-lactamase (ESBL) subsets were interpreted per CLSI criteria.

**Results:**

Gepotidacin (MIC_50_/_90_, 4/16 mg/L) displayed activity against 2,001 *K. pneumoniae* isolates (Table), with 94.9% of all observed gepotidacin MICs ≤ 16 mg/L. Susceptibility rates of oral comparators tested were ≤ 90.0% for amoxicillin-clavulanic acid (90.0%, MIC_50/90_, 2/8 mg/L), ciprofloxacin (86.3%, MIC_50/90_, 0.015/0.5 mg/L), levofloxacin (88.3%, MIC_50/90_, 0.06/1 mg/L), nitrofurantoin (34.0%, MIC_50/90_, 64/ > 128 mg/L), and trimethoprim-sulfamethoxazole (84.7%, MIC_50/90_, ≤ 0.12/ > 4 mg/L) (Table). Gepotidacin remained active against the 11.0% and 6.8% of *K. pneumoniae* isolates that displayed ESBL and MDR phenotypes, respectively, with observed MIC_50/90_ values of 8/32 mg/L for both.

**Conclusion:**

Gepotidacin demonstrated potent *in vitro* activity against contemporary *K. pneumoniae*, including ESBL-producing and MDR isolates. Almost all oral comparator agents reported against US *K. pneumoniae* UTI isolates had susceptibility rates between 80-90% while the observed nitrofurantoin susceptibility rates were 34.0%, respectively.

**Disclosures:**

**Renuka Kapoor, PhD**, GSK: Employee|GSK: Stocks/Bonds (Public Company) **Didem Torumkuney, PhD**, GSK: Employee|GSK: Stocks/Bonds (Public Company) **Rodrigo E. Mendes, PhD**, GSK: Grant/Research Support

